# Psychobiotics and the Manipulation of Bacteria–Gut–Brain Signals

**DOI:** 10.1016/j.tins.2016.09.002

**Published:** 2016-11

**Authors:** Amar Sarkar, Soili M. Lehto, Siobhán Harty, Timothy G. Dinan, John F. Cryan, Philip W.J. Burnet

**Affiliations:** 1 Department of Experimental Psychology, University of Oxford, Oxford OX1 3UD, UK; 2 Institute of Clinical Medicine/Psychiatry, University of Eastern Finland, FI-70211, Kuopio, Finland; 3 Department of Psychiatry, Kuopio University Hospital, FI-70211, Kuopio, Finland; 4 Department of Psychiatry, University College Cork, Cork, Ireland; 5 Department of Anatomy and Neuroscience, University College Cork, Cork, Ireland; 6 Department of Psychiatry, University of Oxford, Oxford OX3 7JX, UK

**Keywords:** gut–brain axis, interkingdom signalling, microbiome, microbiota, prebiotics, probiotics

## Abstract

Psychobiotics were previously defined as live bacteria (probiotics) which, when ingested, confer mental health benefits through interactions with commensal gut bacteria. We expand this definition to encompass prebiotics, which enhance the growth of beneficial gut bacteria. We review probiotic and prebiotic effects on emotional, cognitive, systemic, and neural variables relevant to health and disease. We discuss gut–brain signalling mechanisms enabling psychobiotic effects, such as metabolite production. Overall, knowledge of how the microbiome responds to exogenous influence remains limited. We tabulate several important research questions and issues, exploration of which will generate both mechanistic insights and facilitate future psychobiotic development. We suggest the definition of psychobiotics be expanded beyond probiotics and prebiotics to include other means of influencing the microbiome.

## Trends

Psychobiotics are beneficial bacteria (probiotics) or support for such bacteria (prebiotics) that influence bacteria–brain relationships.

Psychobiotics exert anxiolytic and antidepressant effects characterised by changes in emotional, cognitive, systemic, and neural indices. Bacteria–brain communication channels through which psychobiotics exert effects include the enteric nervous system and the immune system.

Current unknowns include dose-responses and long-term effects.

The definition of psychobiotics should be expanded to any exogenous influence whose effect on the brain is bacterially-mediated.

## The Microbiome–Gut–Brain Axis

The gut microbiome comprises all microorganisms and their genomes inhabiting the intestinal tract. It is a key node in the bidirectional **gut–brain axis** (see Glossary) that develops through early **colonisation** and through which the brain and gut jointly maintain an organism's health. A pivotal study found that mice raised in sterile environments and therefore lacking indigenous bacteria (germ-free mice) showed exaggerated physiological reactions to stress compared to normal controls. The abnormal reactions were reversible through probiotic-induced bacterial recolonisation [1]. This finding revealed the microbiome's causal involvement in the development of the **hypothalamic–pituitary–adrenal (HPA) axis**. Gut bacteria have since been found to participate in the regulation of varied and important physiological processes, including immunomodulation, adiposity, and energy balance 2, 3, 4, 5 as well as the electrophysiological activity of the **enteric nervous system** 6, 7.

Probiotics, beneficial bacteria that yield positive health outcomes, have received particular attention, both in the popular press and from the research community. Here, we critically evaluate efforts to manipulate **commensal gut bacteria** with psychobiotics. These psychobiotics were first defined as probiotics that, when ingested in appropriate quantities, yield positive psychiatric effects in psychopathology [8]. The bacteria most frequently exploited as probiotics are the Gram-positive *Bifidobacterium* and *Lactobacillus* families 9, 10. *Bifidobacteria* and *Lactobacilli* do not possess pro-inflammatory lipopolysaccharide chains, and so their propagation in the gut does not trigger full-fledged immunological reactions. With the presence of such bacteria, the immune system learns to distinguish to between pro- and anti-inflammatory entities and develops appropriate immunogenic responses by identifying pro-inflammatory elements as antigenic [11]. It should be noted, however, that Gram-positive bacteria are not always beneficial, and some, such as the *Clostridia* family, may be pathogenic.

We propose that the definition of psychobiotics be expanded along two dimensions: First, research on healthy individuals is demonstrating that psychobiotic benefits need not be restricted to clinical groups. Second, we include prebiotics in the definition of psychobiotics. Prebiotics are compounds that, when fermented in the gut, produce specific changes in bacterial composition or activity [12]. Prebiotics support the growth of intrinsic commensal bacteria. The majority of prebiotic compounds examined for their neural effects are fructans and oligosaccharides (comprising three to nine saccharide units).

This review will: (i) discuss psychobiotic effects on emotional, cognitive, systemic, and central processes in animals and humans, in clinical and healthy populations, and (ii) assess the microbiome–brain signalling mechanisms enabling these effects.

## Psychophysiological Effects of Psychobiotics

Much psychobiotic research is based on rodent models, which use **rodent stress inductions** and **rodent behavioural tests** to assess motivation, anxiety, and depression. Psychobiotics applied to rodent models of illness, infection, and neurodegeneration also provide early clinical insight into human diseases (Table 1). Human investigations represent a very recent trend. The psychophysiological effects of psychobiotics fall into the following three categories: (i) Psychological effects on emotional and cognitive processes. (ii) Systemic effects on the HPA axis and the **glucocorticoid stress response**, and **inflammation** which is often characterised by aberrant **cytokine** concentrations. Pro-inflammatory cytokines share a strong and well-studied positive association with psychiatric conditions such as depression [13]. For example, injection of interferon-α, a pro-inflammatory cytokine, has been shown to induce depression, which can be alleviated through antidepressant action 14, 15. (iii) Neural effects on neurotransmitters and proteins. Relevant neurotransmitters include γ-aminobutyric acid (GABA) and glutamate, which control neural excitation–inhibition balance. Proteins include brain-derived neurotrophic factor (BDNF), which plays a crucial role in learning and memory processes, including spatial learning, extinction of conditioned fear, and object recognition 16, 17. BDNF is reduced in anxiety and depression, a reduction that is reversible through antidepressant action [18].

## Rodent Models

### Probiotics

Psychobiotics affect psychophysiological markers of anxiety and depression. One study employed a maternal-separation model to induce early-life stress in infant male Sprague–Dawley rats [19]. Rat pups (*n* = 33) were either undisturbed, or, if separated, administered with either a neutral vehicle substance, an antidepressant (the selective serotonin reuptake inhibitor citalopram), or the probiotic *Bifidobacterium infantis*. Vehicle rats showed typical psychophysiological patterns associated with maternal separation, including poorer performance on the forced swim test and increased inflammation (indexed by heightened peripheral concentrations of the pro-inflammatory cytokine interleukin-6), decreased presence of the neurohormone noradrenaline in the brain, and elevated concentrations of amygdala corticotrophin-releasing factor **messenger ribonucleic acid** (mRNA). In comparison, these indices were normalised in probiotic-fed rats. Moreover, these outcomes were comparable to those observed in the citalopram group, suggesting that some probiotic benefits resemble antidepressant effects. These findings were reminiscent of earlier work [20], where a mixture of *Lactobacillus rhamnosus* R0011 and *Lactobacillus helveticus* R0052 downregulated HPA-axis activity (indexed by normalised corticosterone secretion) and mitigated colonic dysfunction in maternally-separated Sprague–Dawley rat pups (*n* = 7–15/group).

In the case of nonclinical populations, experiments are defined by psychobiotic supplementation in rodents that have unimpaired psychophysiological processes and microbiomes. For example, one study examined the effects of probiotics in healthy adult male BALB/c mice (*n* = 36), which were innately stress-sensitive and anxious but were otherwise healthy [21]. Compared to mice administered a vehicle, those fed *Lactobacillus rhamnosus* JB-1 expressed fewer depressive and anxious behaviours (in the forced swim and elevated plus maze tasks, respectively). These changes were accompanied by a blunted corticosterone response to stress, suggesting that the probiotic downregulated HPA-axis activity. In the brain, probiotics differentially altered expression of inhibitory GABA receptors in a region-dependent manner. For instance, in comparison to controls, the probiotic reduced expression of GABA_B1b_ mRNA in the hippocampus and amygdala but increased its expression in prelimbic and cingulate regions. GABA is the chief inhibitory neurotransmitter in the nervous system. Overall, probiotics may modulate regional excitation–inhibition balance, and these changes may be linked with reductions in anxiety- and depression-related behaviour and associated systemic responses.

In another study, healthy adult male BALB/c mice fed *Mycobacterium vaccae* (*n* = 7–10/group across five experiments) displayed reduced anxiety in a maze-learning task [22]. Furthermore, performance on the maze task was substantially improved in the probiotic-fed mice, which completed the maze faster and with fewer errors, benefits that persisted at 1 week, but not 3 weeks, post-treatment. These results provide preliminary but important evidence of the potential longevity of effects, an area that has received little consideration in psychobiotic research.

One programme of research [23] investigated the effect of *Bifidobacteria infantis* on male Sprague–Dawley rats (*n* = 20) in the forced swim test, stress responses, inflammation, and monoaminergic activity. While there were no behavioural changes in swim test performance, there were significant increases of tryptophan, the serotonin precursor, in the plasma. There were also decreased concentrations of 5-hydroxyindoleacetic acid, the serotonin metabolite, in the brain. This was taken as evidence of reduced serotonergic turnover. Compared to vehicle-fed rats, blood from probiotic-fed rats exhibited reduced concentrations of the pro-inflammatory cytokines tumour necrosis factor-α, interleukin-6, and interferon-γ. These reductions jointly characterise a dampened pro-inflammatory response. This approach highlights the importance of examining physiological variables in psychobiotic research, as physiological changes were noted in the absence of significant behavioural changes.

Probiotic effects during stressful experiences have also been examined [24]. Healthy adult male Sprague–Dawley rats were administered *Lactobacillus helveticus* NS8, citalopram, or no intervention, while exposed to chronic-restraint stress (*n* *=* 24 total across treatment groups, *n* = 8 in an undisturbed group). Relative to the control group, the probiotic-fed rats showed lower levels of post-restraint anxiety (assessed in the elevated plus maze and the open-field test), as well as enhanced post-restraint object-recognition memory. At the biochemical level, probiotic-supplemented rats displayed lower levels of corticosterone and adrenocorticotropic hormone. The probiotic group also showed increases in the anti-inflammatory cytokine interleukin-10, and in hippocampal BDNF mRNA, noradrenaline, and serotonin. Overall, probiotic and antidepressant effects were comparable.

Recent work [25] has also studied psychobiotic-induced changes in central neurotransmitter concentrations *in vivo* using magnetic resonance spectroscopy (MRS). Healthy adult male BALB/c mice (*n* = 28) were administered with either *Lactobacillus rhamnosus* JB-1 or a vehicle for 4 weeks. Probiotic-fed mice showed elevated concentrations of glutamate and glutamine, total *N*-acetyl aspartate + *N*-acetyl aspartyl glutamic acid (tNAA), and GABA. The authors interpreted tNAA changes as a marker of alterations in neural metabolism resulting from the intervention. Glutamate is the chief excitatory neurotransmitter in the central nervous system, and, to the best of our knowledge, this is the first demonstration that it is sensitive to probiotics. The implications of concurrent elevations in both glutamate and GABA for regional excitation-inhibition balance are currently unknown, but are suggestive of an overall metabolic increase. Furthermore, because GABA and glutamate have opposing effects on neural excitability, it is possible that the total psychobiotic effect may be occurring within a zero-sum framework. The researchers also conducted periodic MRS, finding differential rates of emergence for the effects. For example, NAA increased after 2 weeks of probiotics, an elevation that was sustained for the remainder of the supplementation, and which returned to baseline 4 weeks thereafter. Glutamate and glutamine levels also increased after 2 weeks, and then remained elevated for a further 6 weeks, including 4 weeks after the intervention. Finally, GABA concentrations were only elevated in the fourth week of the intervention, but not before or after. These results represent a crucial step towards determining emergence and longevity of effects. While the general consensus is that ingestion of probiotics results in transient, rather than permanent, colonisation of the gut [26], these findings suggest both that psychobiotics may have some long-term effects, and that the effects have differential longevity.

### Prebiotics

A much smaller number of studies has examined the psychophysiological effects of prebiotics. These include investigations of galacto-oligosaccharides (GOS) and fructo-oligosaccharides (FOS), which are a source of nutrition for *Bifidobacteria* and *Lactobacilli*, and stimulate their activity and propagation in the gut. The first report of the psychobiotic properties of prebiotics examined adult male Sprague–Dawley rats (*n* = 24) that were administered the Bimuno formulation of GOS (B-GOS), FOS, or water, over 5 weeks [27]. Relative to controls, prebiotic ingestion increased hippocampal BDNF expression and BDNF mRNA expression in the dentate gyrus. Prebiotic feeding also increased *N*-methyl-d-aspartate receptor (NMDAR) subunits expressed in the hippocampus. These receptors play an essential role in maintaining synaptic plasticity and optimal memory function [28]. Both B-GOS and FOS elevated NR1 subunit expression in the hippocampus, with B-GOS additionally increasing NR2A subunits in this region, and NR1 and d-serine in the frontal cortex. The more widespread B-GOS effect, relative to FOS, may reflect the former's greater *Bifidogenic* capacity. There is also evidence of substantial benefits conferred by the human milk oligosaccharide 2′-fucosyllactose [29]. Relative to vehicle, male rodents (both Sprague–Dawley rats and C57BL/6 mice) showed enhanced associative learning and working memory, as well as higher expression of hippocampal and striatal BDNF and increased hippocampal long-term potentiation.

Prebiotic supplementation has also been studied in neonatal rats [30]. Male and female Sprague–Dawley rat pups (*n* = 48) were fed daily with B-GOS or a control solution from post-natal day 3 to 21. Animals supplemented with B-GOS expressed higher levels of hippocampal BDNF and NMDAR subunit GluN2A. Crucially, these changes were observed even 26 days after treatment cessation. A similar effect of human milk oligosaccharide was observed in male Lister Hooded rat pups (*n* = 60) [31]. Relative to vehicle, rats fed the prebiotic during lactation showed substantially enhanced maze-learning and object-recognition one year later. These findings have important implications for assessing the longevity of prebiotic effects, and are suggestive of very long lasting gains.

## Human Research

### Probiotics

The rodent-human translation has been surprisingly robust, though many more human studies are necessary. In an important early investigation [32], male and female participants (*n* = 124) consumed either a fermented milk drink containing *Lactobacillus casei Shirota* or a placebo. At the end of the 3-week intervention, there were no overall changes in self-reported affect. However, when only participants whose baseline mood scores fell in the lowest third of the total range were analysed, probiotic supplementation resulted in significantly more participants self-rating as happy rather than depressed, relative to placebo. These results suggest that the emotional benefits of psychobiotics may be subject to ceiling effects. The researchers also found that the probiotic-fed participants performed lower on two assessments of memory function. This may be attributable to chance, as the authors themselves have suggested, but it may also imply possible detrimental effects of psychobiotics.

Another well-known study provided evidence of improved mood in a generally healthy sample [33]. In a randomised and double-blind design, healthy male and female volunteers (*n* = 55) consumed either a mixture of probiotics (*Lactobacillus helveticus* R0052 and *Bifidobacterium longum*) or a placebo over 30 days, after which participants completed a range of self-report measures on mood and distress. Participants also collected urine over 24 hours before and after the intervention, enabling cortisol estimations. Relative to placebo, probiotic-treated participants showed significant declines in self-reported negative mood and distress. Parallel to these changes was a decrease in urinary free cortisol, which is suggestive of reduced stress. Interestingly, a follow-up analysis of the individuals with the lowest stress (indexed by cortisol concentrations) showed similar affective benefits to those with higher cortisol concentrations [34], to some extent contravening the role of ceiling effects in determining psychobiotic outcomes. The researchers also investigated potential detrimental effects, including probiotic-induced impairments in learning and memory. However, there was no evidence of dysfunctions in learning and memory, and furthermore, the probiotics did not induce addiction, suggesting a good safety profile without concomitant cognitive impairments.

Similar effects have been observed in other investigations of mood. For instance, in a recent randomised controlled trial [35], healthy male and female participants (*n* = 40) consumed either a placebo product or a mixture of several probiotics (*Bifidobacterium bifidum* W23, *Bifidobacterium lactis* W52, *Lactobacillus acidophilus* W37, *Lactobacillus brevis* W63, *Lactobacillus casei* W56, *Lactobacillus salivarius* W24, and *Lactococcus lactis* W19 and W58) over a period of 4 weeks. Relative to placebo, probiotic-treated participants exhibited substantially reduced reactivity to sad mood (assessed by the Leiden Index of Depression Sensitivity Scale), an effect that was specifically attributable to reduced rumination and aggressive cognition.

*Lactobacillus casei Shirota* has also been recently employed in an intriguing study of academic stress [36]. Healthy male and female students (*n* = 47) consumed either the probiotic or a placebo for 8 weeks before a medical school examination. Physiological measures were obtained for this duration and after the examination as well. The probiotic group had substantially lower plasma cortisol compared to the placebo group on the day before the examination. Two weeks post-examination, the probiotic group showed significantly higher faecal serotonin, though the psychological implications of this change are less clear. Another study found that, relative to placebo, student athletes (*n* = 44) fed *Lactobacillus gasseri* OLL2809 LG2809 showed elevated mood and reduced natural killer cell activity after strenuous exercise, with some additional alleviation of fatigue when the probiotic was consumed alongside α-lactalbumin [37]. These results suggest that probiotics may have ecologically relevant benefits and the potential to enhance performance on some important life activities.

Some evidence for the immunological effects of probiotics in humans derives from a study in individuals with irritable bowel syndrome [38], which is associated with disturbances in the gut–brain axis [39] and in the composition of the microbiome [40], and is often accompanied by anxiety and depression [41]. Male and female participants (*n* = 77) consumed either *Lactobacillus salivarius* UCC4331, *Bifidobacterium infantis* 35624, or a placebo. At baseline, participants had an aberrant ratio of interleukin-10 to interleukin-12, suggesting a generalised pro-inflammatory state. Only those participants who consumed *Bifidobacterium infantis* 35624 displayed a normalisation of this ratio post-treatment. These results indicate both that probiotics can induce cytokine changes in humans, and also that these effects may be specific to particular families or strains of probiotic. However, there is no theoretical basis at present to predict that one form of probiotic would be more effective than another.

What neural and information-processing changes might underpin these probiotic-induced emotional benefits in humans? Evidence from a neuroimaging study points to a modulation of attention and vigilance to negative emotional stimuli [42]. Over 4 weeks, healthy female participants consumed either a placebo or a mixture of probiotics (*Bifidobacterium animalis*, *Streptococcus thermophiles*, *Lactobacillus bulgaricus*, and *Lactococcus lactis*), or consumed nothing as part of a passive control (total *n* = 36). Crucially, participants underwent functional magnetic resonance imaging (fMRI) to determine how probiotic ingestion affected neurophysiological activity. During image acquisition, participants were shown emotional faces that are known to capture attention and cause brain activation, fearful faces in particular [43]. Relative to placebo, probiotic-treated participants showed decreased activity in a functional network associated with emotional, somatosensory, and interoceptive processing, including the somatosensory cortex, the insula, and the periaqueductal gray. Placebo participants showed increased activity in these regions in response to emotional faces. This can be interpreted as a probiotic-induced reduction in network-level neural reactivity to emotional information.

### Prebiotics

Inductive evidence that psychobiotics modulate emotional appraisal is supplied by the first human study to examine the psychophysiological effects of prebiotics [44]. Healthy male and female participants (*n* = 45) consumed either B-GOS, FOS, or a placebo. In comparison to the other two groups, participants who consumed B-GOS showed a significantly reduced **waking-cortisol response**. Exaggerated waking cortisol is a biomarker of emotional disturbances such as depression 45, 46. Furthermore, participants completed an emotional dot-probe task that measures vigilance, or attention to negative stimuli, which is also a behavioural marker of anxiety and depression [47]. B-GOS attenuated vigilance, suggestive of reduced attention and reactivity to negative emotions. Attenuated vigilance is considered an anxiolytic and antidepressant effect [48].

Overall, then, psychobiotics may exert their beneficial effects on mood through modulation of neural networks associated with emotional attention. The addition of behavioural measures of vigilance, cognitive control, and negative mood to research programmes would richly supplement self-reports. Moreover, their addition is logistically straightforward and incurs minimal additional resources. Reduced attention to negative stimuli may constitute a neurocognitive channel through which psychobiotics improve mood. At the systemic level, reductions in cortisol and pro-inflammatory cytokines would support these processes, given their frequent co-occurrence with negative mood. At present, however, the direction of causality between systemic and brain changes is unknown. Furthermore, longevity and time-courses of effects have not been studied in humans and are even less clear than in rodents.

## Bacteria–Brain Signalling

**Figure 1:**
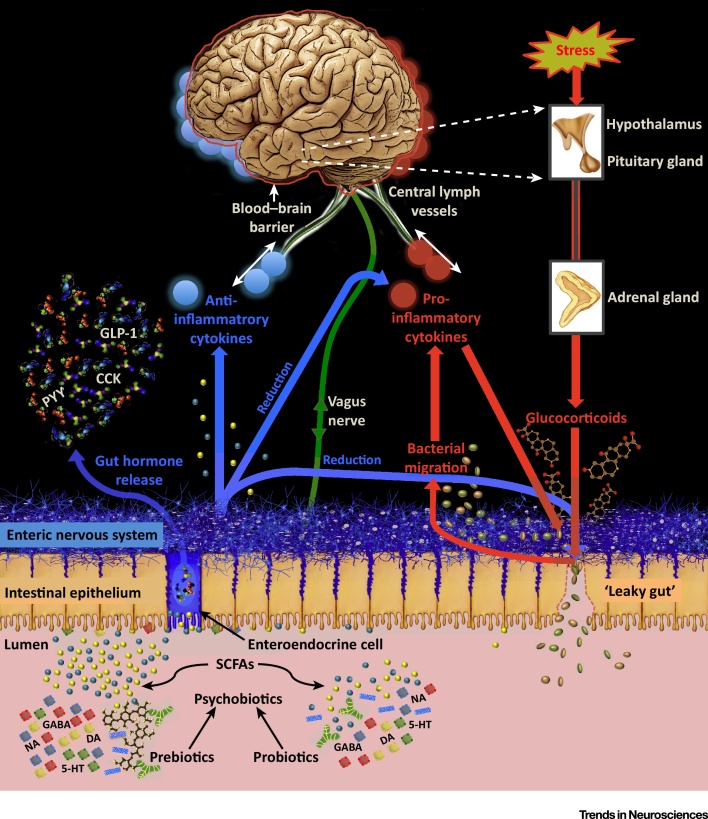
Systems-Level Overview of Psychobiotic Action. Blue arrows indicate psychobiotic processes and effects, while red arrows indicate processes associated with leaky gut and inflammation. Probiotics directly introduce beneficial bacteria such as *Lactobacilli* and *Bifidobacteria* into the gut. Prebiotics (e.g., galacto-oligosaccharides) support the growth of such bacteria. **SCFAs and gut hormones**: Both probiotics and prebiotics increase production of short-chain fatty acids (SCFAs), which interact with gut mucosal enteroendocrine cells and catalyse the release of gut hormones such as cholecystokinin (CCK), peptide tyrosine tyrosine (PYY) and glucagon-like peptide- 1 (GLP-1). Prebiotics may have stronger effects in this regard in comparison to probiotics. SCFAs and gut hormones enter circulation and can migrate into the central nervous system. Gut hormones are also secreted by tissues other than enteroendocrine cells. **Neurotransmitters**: psychobiotics enhance neurotransmitter production in the gut, including dopamine (DA), serotonin (5-HT), noradrenaline (NA), and γ-aminobutyric acid (GABA), which likely modulate neurotransmission in the proximal synapses of the enteric nervous system. **Vagal connections**: the vagus nerve synapses on enteric neurons and enables gut–brain communication. **Stress, barrier function, and cytokines**: barrier dysfunction is exacerbated through stress-induced glucocorticoid exposure. This enables migration of bacteria with pro-inflammatory components, increasing inflammation directly and also triggering a rise in pro-inflammatory cytokines via the immunogenic response. These cytokines impair the integrity of the blood–brain barrier and permit access to potentially pathogenic or inflammatory elements. Pro-inflammatory cytokines (red circles) also reduce the integrity of the gut barrier. Psychobiotic action restores gut barrier function and decreases circulating concentrations of glucocorticoids and pro-inflammatory cytokines. They also increase concentrations of anti-inflammatory cytokines (blue circles), which enhance integrity of the blood–brain barrier, the gut barrier, and reduce overall inflammation. Cytokines clustering at the brain represent cytokine interaction with the blood–brain barrier. **Central lymphatic vessels**: cytokines may interact more directly with the brain than previously appreciated through the recently discovered central lymphatic vessels.

The mechanisms through which psychobiotics exert their effects have yet to be clearly defined and remain poorly understood. Though there are some studies that provide mechanistic insights for humans, the majority of research is based on rodent models. A crucial step in developing knowledge of the mechanisms lies in investigating how the microbiome and the brain communicate with one another (see Figure 1).

### Bacteria–Enteric Nervous System Interactions

Gut bacteria regulate electrophysiological thresholds in enteric nervous system neurons. For example, myenteric neurons exposed to *Bifidobacterium longum* NCC3001-fermented substances showed reduced generation of action potentials when they were electrically stimulated [49]. Similarly, colonic AH neurons (the chief sensory neurons in the colon) treated with *Lactobacillus rhamnosus* showed increased excitability, an effect that emerged from inhibition of calcium-controlled potassium gates [50]. Other work showed that neurons from the dorsal root ganglion in the colon did not display hyperexcitability in response to noxious stimulation if they had been treated with *Lactobacillus rhamnosus* 51, 52. Myenteric neurons are also in close proximity to the gut lumen [7], which would facilitate their contact with the microbiome. In germ-free mice, these neurons show lower levels of excitability compared to their normally-colonised counterparts [53]. One study found evidence of intestinal neural abnormalities in the jejunum and ileum of germ-free mice in comparison to controls [54], with germ-free mice showing reduced nerve density, fewer nerves per ganglion, and a greater number of myenteric nitrergic neurons. Recent evidence also indicates that the microbiome affects ion transport controlled by cyclic adenosine monophosphate (cAMP) [55].

Overall, these results provide striking evidence of direct, bacteria-induced modulation of the enteric nervous system. Moreover, the influence of the microbiome on the enteric nervous system extends beyond neurons, with recent findings demonstrating that gut bacteria also play a crucial role in the development and homeostasis of glial populations in the gut [56].

Gut bacteria also produce a range of neurotransmitters through the metabolism of indigestible fibres. These include dopamine and noradrenalin by members of the *Bacillus* family, GABA by the *Bifidobacteria* family, serotonin by the *Enterococcus* and *Streptococcus* families, noradrenalin and serotonin by the *Escherichia* family, and GABA and acetylcholine by the *Lactobacilli* family 57, 58, 59. Though there is no direct evidence as of yet, it is likely that these neurotransmitters modulate synaptic activity in the proximal neurons of the enteric nervous system, and is an important avenue for future research.

### Vagal Signalling

The vagus nerve plays an essential and wide-ranging role in coordinating parasympathetic activity, including regulation of heart rate and gut motility. It possesses an abundance of sensory fibres, and is able to convey rich information on organ function throughout the body to the brain [60]. Vagal activity is sensitive to nutrition, exercise, and stress 61, 62, 63.

Stimulating the vagus nerve exerts anti-inflammatory effects [64], and is used therapeutically for refractory depression, pain, and epilepsy 65, 66, 67, 68. There is also evidence of both antidepressants and anxiolytics exerting vagal effects 69, 70, 71, suggesting that vagal modulation may be a common pathway for the effects of antidepressants, anxiolytics, and psychobiotics.

Several animal studies have found that the vagus nerve mediates the relationship between psychobiotics and their psychophysiological effects, as severing the vagus nerve (vagotomy) abolishes responses to psychobiotic administration 21, 49, 72. However, one study has found that ingestion of antimicrobials increased intrinsic relative abundance of *Lactobacilli* in innately anxious male BALB/c mice, a change that was accompanied by increased exploratory behaviour and BDNF expression. Crucially, however, vagotomy did not eliminate these neural or behavioural benefits [73]. Therefore, vagal signalling may be at most a partial mediator of bacterial effects.

### Short-Chain Fatty Acids, Gut Hormones, and Bacteria-Derived Blood Metabolites

The human gut is incapable of digesting macronutrients such as plant polysaccharides. While these frequently appear in the diet, the human genome does not code the requisite enzymes for their digestion, which are supplied by the microbiome [74]. The metabolisation of these fibres produces short-chain fatty acids (SCFAs), including acetate, butyrate, lactate, and propionate 75, 76. SCFAs enter the circulatory system through the large intestine [77], where the greater proportion are directed into the liver and muscle. Although it is unclear to what extent the small fraction of SCFAs crossing into the central nervous system modulates neurotransmission, there is some evidence for their psychotropic properties at pharmacological concentrations. For instance, systemic sodium butyrate injections (200 mg/kg body weight) in rats produce antidepressant effects, and increase central serotonin neurotransmission and BDNF expression [78]. Here, the action of butyrate as an epigenetic modifier [79] is more likely compared to action as an agonist at a free fatty acid receptor (FFAR), given that there are few FFARs in the brain [80]. However, it should be noted that the SCFAs display pleiotropy (independent effects produced by a single gene), and also stimulate the HPA axis [81] or have direct effects on the mucosal immune system [82], which may indirectly affect central neurotransmission. A recent rodent investigation [83] has also found that the SCFA acetate plays a causal role in obesity. Acetate generated by the gut bacteria in response to high-fat diets triggers parasympathetic activity and promotes increases in ghrelin, glucose-stimulated insulin, and further nutrition intake, creating a positive feedback loop that increases the likelihood of obesity.

SCFAs also influence secretion of satiety peptides, including cholecystokinin (CCK), peptide tyrosine tyrosine (PYY) and glucagon-like peptide-1 (GLP-1), from gut mucosal enteroendocrine cells which express FFARs [84]. For instance, propionic acid mediates the release of GLP-1 and PYY through activation of FFAR2 [85]. Consistent with the concept that SCFAs are produced from the bacterial metabolism of dietary polysaccharides, prebiotic supplementation increases the production of intestinal SCFAs, which modulate enteroendocrine cells and their secretion of PYY and GLP-1 77, 86. It is therefore reasonable that the satiety hormones may play a more significant role in the central effects of prebiotics compared to probiotics. Furthermore, circulating PYY and GLP-1 have brain-penetrant properties, and their administration to rodents have significant effects on neurotransmitters and behaviour 87, 88, 89.

The microbiome has also been shown to possess a substantial role in generating metabolites that enter circulation and exert a range of consequences outside the gut [90]. A key study that compared germ-free mice to normally colonised mice found striking effects of the microbiome on the diversity and quantity of blood metabolites [91]. For instance, germ-free mice had 40% greater plasma tryptophan concentrations than normal mice, but the normal mice had 2.8 times greater plasma serotonin levels than the germ-free mice. This suggests that gut bacteria crucially affect the metabolism of tryptophan into serotonin in Enterochromaffin cells (serotonin-secreting cells embedded in the luminal epithelium). Though the specific mechanism through which bacteria might control serotonin production in Enterochromaffin cells was unknown at that time, a recent study has attributed this role to indigenous spore-forming bacteria in the gut [92]. There were similarly dramatic differences in other tryptophan metabolites, especially those containing indole, such as the antioxidant indole-3-propionic acid (IPA) and indoxyl sulphate, which were undetected in the germ-free mice and whose production was therefore interpreted as being fully mediated by gut bacteria.

We speculate that these metabolites are sensitive to psychobiotic action. However, the relationships between the microbiome, bacteria-derived metabolites, and the central nervous system, as well as the role of psychobiotics in modulating this network, remain virtually unexplored.

### Bacteria-Immune Interactions

A key function of the immune system is to detect and eliminate pathogens. Every microbe possesses a microbe-associated molecular pattern (MAMP, previously referred to as pathogen-associated molecular patterns) [93]. A range of microscopic elements may act as MAMPs, including microbial nucleic acids, molecular cell wall components (e.g., lipopolysaccharides), or bacterial flagella. Gut microbes can communicate with the enteric nervous system and the innate immune system via interactions between the MAMPs and pattern-recognition receptors embedded along the lumen. The family of pattern-recognition receptors includes Toll-like receptors (TLRs), C-type lectins, and inflammasomes. These receptors are able to detect the nature and potential effects of various microbes via the MAMPs and, at a broad level, transmit information about the microbial environment to the host, enabling specific immunological responses.

The MAMPs of beneficial bacteria, by triggering pattern-recognition receptors, may precipitate secretion of anti-inflammatory cytokines such as interleukin-10 38, 94. While rigorous mechanistic descriptions of the relationship between MAMPs, pattern-recognition receptors, and reductions in inflammation are lacking, one intriguing hypothesis is that beneficial bacteria might serve as physical barriers that block pathogenic MAMPs (e.g., lipopolysaccharides) from activating host pattern-recognition receptors such as TLR2 and TLR4 by binding to them instead, thereby preventing pro-inflammatory responses [95].

Prebiotics may act in a similar capacity, as there is evidence of direct interaction between oligosaccharides and the epithelium, independent of gut bacteria, with substantial reductions in pro-inflammatory cytokines 96, 97. Prebiotics may prevent pathogenic MAMPs from accessing pattern-recognition receptors, either by acting as physical barriers to reduce the incidence of MAMP binding, or by directly binding to the receptor themselves. Thus, prebiotics need not exert all of their beneficial effects exclusively by growing commensal bacteria.

One mechanism for psychobiotic effects is the mitigation of low-grade inflammation, typically observed as reductions in circulating pro-inflammatory cytokine concentrations. Pro-inflammatory cytokines are also capable of increasing the permeability of the blood–brain barrier [98], permitting access to potential pathogenic entities. Cytokines alter concentrations of several neurotransmitters that regulate communication in the brain, including serotonin, dopamine, and glutamate [99]. Cytokines can also enter the brain through active uptake, stimulating secretion of pro-inflammatory substances such as prostaglandins [100], precipitating further inflammation. There is also emerging evidence of a lymphatic drainage system subserving the brain [101], which we speculate may allow cytokines to interact with neural tissue.

A parallel mechanism underlying psychobiotic-induced reductions in inflammation is the increase of anti-inflammatory cytokines such as interleukin-10. For example, in humans, *Bifidobacterium infantis* 35624 and *Lactobacillus GG* 38, 102 have been shown to enhance concentrations of interleukin-10. By reducing the total quantity of pro-inflammatory cytokines, either directly or by increasing anti-inflammatory cytokines, psychobiotics may be reducing the probability of cytokines gaining access to the central nervous system, and may also be restoring inflammation-induced permeability of the blood–brain barrier. Cytokine interactions at the blood–brain barrier are highly complex and more detailed discussions of those mechanisms are beyond the scope of this review. The reader is referred to existing research in this area 103, 104, 105.

A parasitic infection study [106] yielded an important mechanistic insight regarding cytokine roles in microbiome–brain signalling. Healthy male AKR mice were infected with the *Trichuris muris* parasite, following which they were treated with *Bifidobacterium longum* NCC3001*, Lactobacillus rhamnosus* NCC4007, or vehicle. Infection increased anxious behaviour and reduced hippocampal BDNF mRNA levels. *Bifidobacterium longum* NCC3001 (but not *Lactobacillus rhamnosus* NCC4007) reduced anxious behaviour and normalised BDNF mRNA concentrations. However, these changes occurred in the *absence* of prebiotic-induced reductions in any pro-inflammatory cytokines. This may be interpreted as evidence that psychobiotic effects also occur through mechanisms other than cytokine reduction.

Pro-inflammatory cytokines are also known to compromise the integrity of the gut barrier 107, 108. For example, *Lactobacillus rhamnosus* GG ameliorates gut barrier dysfunction by inhibiting the signalling potential of pro-inflammatory cytokines such as tumour necrosis factor-α [109].

The microbiome also plays a substantial role in broader immunological functions. For example, the presence of bacteria such as *Bifidobacterium infantis*35624 and *Lactobacillus salivarius* UCC118 in the gut has been shown to affect immunogenic responses toward pathogenic entities such as *Salmonella typhimurium* [110]. The microbiome also contributes to the development of the immune system. For example, the expression of colonic effector pro-inflammatory genes that are sensitive to the action of interleukin-10 is bacteria-dependent [111]. Exogenous bacteria can also trigger the development of immunogenic responses under certain conditions. For instance, the introduction of *Helicobacter hepaticus* in the presence of genetically-deficient interleukin-10 signalling systems led to an increase in pro-inflammatory marginal zone B cells (in the category of white blood cells, expressing antibodies) of the spleen [112]. The microbiome also controls the development of appropriate immunosuppression in response to dietary antigens through the production of immunosuppressive regulatory T-cells [113]. These cells prevent full immunogenic reactions to normal nutritional input, whereas germ-free mice do not possess this immunosuppressive activity and show exaggerated immune responses to dietary antigens. These bacteria–immune interactions illustrate boundary conditions for psychobiotics. For instance, certain genetic abnormalities that alter the immune system may result in unexpected psychobiotic effects.

### Glucocorticoids and the Gut Barrier

Though stress is not a signalling pathway as such, it nonetheless constitutes an important influence on structural and functional aspects of the microbiome [114]. Glucocorticoids (e.g., cortisol, corticosterone) dysregulate gut barrier function, reducing the integrity of the epithelium and permitting outward migration of bacteria [115], triggering inflammatory immune responses. Bacterial migration outside the lumen could also directly modulate inflammation by raising the concentrations of pro-inflammatory cell elements such as lipopolysaccharide 115, 116, 117, a process associated with human depression 117, 118. Probiotic supplementation with the *Bifidobacterium* or *Lactobacillus* families is able to restore gut-barrier integrity and reduce stress-induced gut leakiness in mice and rats 119, 120.

However, both effects on glucocorticoids and cytokines as mechanisms of action for psychobiotic-induced benefits follow ceiling-effect logic. These are reasonable mechanisms for therapeutic benefits in cases of inflammation, stress, or poor gut-barrier function at baseline, but cannot explain the benefits observed in healthy groups where these abnormalities are presumably absent 33, 42, 44.

## Future Directions and Psychobiotics beyond Prebiotics and Probiotics

Narrative reviews of psychobiotics, including this one, are largely enthusiastic about the field. However, a recent systematic review of psychiatric benefits of probiotics in humans found little evidence of positive outcomes [121], a finding running counter to the general optimism. Others have stated that the field, though not ‘faecal phrenology’, will ultimately be unable to provide true translational value without rigorous elucidation of mechanisms [122]. Numerous limitations must both constrain enthusiasm and stimulate further investigations. For example, many studies examine several psychophysiological variables, of which only a few register effects. While exciting, issues of false-positives and false-negatives have not been adequately investigated. To a large extent, greater statistical power will add resolution. However, a range of conceptual and technical issues require exploration, which will both provide further mechanistic insights and pave the way for the emergence of systematic and efficient psychobiotics (Table 2).

It is also worthwhile considering a wider definition of psychobiotics that need not be limited to probiotics and prebiotics. Indeed, any substance that exerts a microbiome-mediated psychological effect is potentially a psychobiotic, or at least possesses psychobiotic properties. For example, ingestion of the antipsychotic olanzapine has been shown to increase relative abundance of *Actinobacteria* and *Proteobacteria*, and is associated with weight gain [123]. However, a mixture of antibiotics (neomycin, metronidazole, and polymyxin) has also been shown to ameliorate the effects of the olanzapine on the relative abundance of bacterial families and concomitant weight gain in rats [124]. Antibiotic mixtures (e.g., bacitracin, neomycin, and pimaricin) have been shown to induce neurochemical and behavioural changes through effects on the microbiome [73], and chronic ingestion of antibiotics can permanently alter microbiome composition and metabolism [125]. Therefore, both antibiotics and antipsychotics may also be classified as psychobiotics. Antibiotic and antipsychotic effects on commensal bacteria 123, 124, 125 illustrate the importance of considering the microbiome in side-effects assessments during clinical trials, which is currently not on the research agenda. Indeed, many substances may exert secondary psychobiotic effects through the microbiome alongside their primary intended effects. Some of these areas are being explored in the emerging field of pharmacomicrobiomics [126]. Beyond medical signals, the microbiome is sensitive to diet 83, 127, 128 and exercise [129], both of which affect mood and cognition, and both of which affect vagal activity 61, 130, 131, therefore sharing a signalling mechanism with other psychobiotics. It is possible the psychological effects of diet and exercise are partially mediated by the microbiome, and in this case, an argument may be made for them possessing psychobiotic properties.

More broadly, and consistent with cultural variance in diet and environment, there are geographical differences in the microbiome, both in infancy and adulthood 132, 133, 134.

Examining current unknowns (see Table 2 and Outstanding Questions) and expanding the ‘psychobiotic’ label should be prioritised to maximally exploit bacteria–brain relationships.

Outstanding Questions What are the dose-response functions associated with psychobiotics?

What are the contributions of gut hormones in the mechanisms of action of prebiotics versus probiotics?

How do prebiotics and probiotics differ in terms of their impact on microbiome structure and relative abundance?

Are there undetected psychophysiological costs alongside the observed benefits of psychobiotics?

Does the brain adapt to long-term psychobiotic ingestion?

How do bacteria-derived blood metabolites affect the central nervous system, and how do psychobiotics modulate this relationship?

What is the time-course for emergence of various psychobiotic effects, and how long do they last?

Are there ceiling effects on psychobiotic benefits?

What are the functional implications of altered excitation–inhibition balance (due to alterations in GABA and glutamate concentrations) in specific brain regions?

Why do some strains of probiotic or prebiotic show effects while others do not, and are these linked to dosage?

Do neurotransmitters produced by gut bacteria modulate synaptic transmission in the proximal neurons of the enteric nervous system?

What is the direction of causality between systemic and central changes?

How do factors such as diet, genotype, sex, and age moderate the effects of psychobiotics?

## Figures and Tables

**Table 1:** Psychobiotics in Rodent Models of Dysfunction

Model	Induction	Psychobiotic	Species	Effects relative to comparison groups	Refs
Alzheimer's disease	A β_1–42_-induced neurotoxicity	Prebiotic, chitosan oligosaccharide	Male Sprague–Dawley rats (*n* = 12)	↑ Cognitive function (Morris water maze), ↓ pro-inflammatory cytokines (tumour necrosis factor-α, interleukin-1β)	[135]
Amyotrophic lateral sclerosis	High level of mutant human SOD1^G93A^ gene	Prebiotic, galacto-oligosaccharides	Male transgenic ALZ mice (*n* = 12)	↓ Motor neuron death, ↓ muscular atrophy, ↑ serum folate, ↑ vitamin B12, ↑ homocysteine	[136]
Autism spectrum disorder	Maternal immune activation	Probiotic*, Bacteroides fragilis*	Offspring of pregnant C57BL/6N mice (*n* = 9–75/group)	↑ Intestinal permeability, ↓ pro-inflammatory cytokines (interleukin-6), ↓ anxiety (open field test), ↓ repetitive behaviour (marble burying), ↑ communication (calling), ↑ sensorimotor gating (startle inhibition)	[137]
Bacterial infection	*Citrobacter rodentium*–induced colitis	Probiotic *Lactobacillus rhamnosus* R0011 + *Lactobacillus helveticus* R0052	Female C57BL/6 mice (*n* = 4–16/group)	↑ Gut barrier function, ↓ transcription of pro-inflammatory cytokines (tumour necrosis factor-α and interferon-γ, interleukin-17), ↑ transcription of anti-inflammatory cytokines (interleukin-10), normalisation of microbiome	[138]
Bacterial infection	*Citrobacter rodentium*–induced colitis	Probiotic *Lactobacillus rhamnosus* R0011 + *Lactobacillus helveticus* R0052	Neonatal C57BL/6 mice (*n* = 4–27/group)	↓ Infection-induced death, ↓ infection-induced weight loss	[139]
Bacterial infection	*Citrobacter rodentium*–induced colitis	Probiotic *Lactobacillus reuteri*	Male CD1 mice (*n* = 105, experiment 1; *n* = 66, experiment 2)	↓ Stress-induced gut-to-spleen pathogen migration	[140]
Bacterial infection	*Citrobacter rodentium*–induced colitis	Probiotic *Lactobacillus reuteri*	Male C57BL/6 mice (*n* = 9/group)	↓ Stress-induced infectious colitis	[141]
Diabetes	Streptozotocin injection	Probiotics, *Lactobacillus acidophilus* + *Bifidobacterium lactis* + *Lactobacillus fermentum*	Male Wistar rats (*n* = 10/group)	↑ Cognitive function (Morris water maze), ↑ hippocampal long-term potentiation (LTP)	[142]
Diabetes	Streptozotocin injection	Probiotics, *Lactobacillus brevis* DPC 6108	Male Sprague–Dawley rats (*n* = 10–15/group)	↓ Glucose, ↓ hyperglycaemia	[143]
Hyperammonemia	Ammonium acetate injection	Probiotic, *Lactobacillus helveticus* NS8	Male Sprague–Dawley rats (*n* = 6/group)	↓ Inflammation (brain-inducible nitric oxide synthase, prostaglandin E2, and interleukin-1β), neurotransmitter processing (↓ abnormal metabolisation of serotonin into 5-hydroxyindole acetic acid), ↓ anxiety (elevated plus maze), ↑ cognitive function (Morris water maze)	[144]
Post-inflammatory anxiety	Lipopolysaccharide injection	Prebiotic, Bimuno-galacto-oligosaccharides (B-GOS)	Male CD1 mice (*n* = 15/group)	↓ Pro-inflammatory cytokines (interleukin-1β), ↓ cortical 5-HT2A receptors	[145]

**Table 2:** Conceptual and Technical Knowledge Gaps in the Development of Psychobiotics

Knowledge Gap	Relevance/Central Question	Suggestive Evidence	Required Investigations
Ecosystem and structural change	Do psychobiotics alter the architecture of the microbiome? Do probiotics and prebiotics differ in this regard?	Current data suggest that probiotics do not modulate the relative abundance of bacterial communities 26, 42, 146, 147. However, these are only short-term studies. There are no long-term investigations, and also no investigations of prebiotic effects.	Longer-term administration of probiotics (e.g., several months), followed by analysis of faecal samples for estimates of gut bacteria concentrations. Prebiotic-induced changes may emerge at a different rate compared to probiotics, owing to different effects on the microbiome, and should be studied separately.
Age	Do psychobiotics exert age-specific effects, given impaired-homeostatic integrity of the microbiome in ageing individuals?	Young and elderly adults have different microbiotic architectures [148]. Microflora differ between individuals who experience healthy ageing and those who do not [149]. In both ageing rats and humans, probiotic administration altered relative abundance of gut bacteria such as increasing *Actinobacteria*, *Bacterioidetes*, and *Lactobacillus* concentrations and decreasing *Clostridium difficile* concentrations 150, 151, 152 and may also ameliorate age-related cognitive deficits in rodents [150].	Systematic comparisons of young and ageing test subjects in response to identical psychobiotic regimes.
Dose response functions	Are psychobiotic effects dose-sensitive?		Experiments in which psychobiotics are administered at varying concentrations, followed by a comparison of the outcomes in each group along the same measures. Certain psychobiotics which seem to have no effect at a given concentration may exert effects further along the dose-response curve.
Time-course of emergence of effects	How long after the beginning of ingestion do psychobiotic effects emerge?	There is initial evidence of differential rates of emergence for various psychobiotic effects. Glutamate rose after 2 weeks of *Lactobacillus rhamnosus* JB-1, but GABA rose after 4 weeks [25]. Future research should examine whether manipulating dose alters the emergence of effects.	Systematic measurement of task performance and physiological changes to determine the time points at which behavioural and biological effects emerge.
Long-term effects	Do psychobiotics produce long-term changes in the central nervous system?	Both probiotics 22, 25 and prebiotics 30, 31 have been shown to induce effects that outlast the period of ingestion, and have implications for the longevity of treatment outcomes.	Systematic tracking of psychological, neural, and systemic changes both during long-term psychobiotic ingestion and after cessation of the regime.
Zero-sum effects	Are changes in one brain region broadly offset by changes in the opposing direction elsewhere?	Very preliminary evidence for a zero-sum effect demonstrating an increase in both GABA *and* glutamate expression [25], which control neural inhibition and excitation, respectively. Earlier research [21] found that probiotics increased GABA representation in some areas but decreased it in others. Overall, this pair of results suggests that changes in excitation–inhibition balance in one brain region may be offset by those in another.	Studies focussed specifically on this type of change (e.g., increase in both GABA and glutamate, or increase of GABA in one area but decrease in another). Research should also follow up the functional implications of these alterations, which may be fundamental in predicting detriments and enhancements.
Cognitive enhancement	Can psychobiotics confer cognitive benefits?	There is yet no evidence of psychobiotic-induced cognitive enhancements in humans. Numerous rodent studies discussed here show evidence of improvement in learning and memory following psychobiotic ingestion 21, 22, 29, 31.	Measurement of memory and learning performance in humans alongside physiological measures. Lack of evidence of enhancement may be due to the tasks themselves not having the sensitivity to detect subtle changes in performance.
Detrimental effects	Are psychobiotic benefits accompanied by undetected costs?	One study found reduced performance on memory tasks following psychobiotic consumption in humans [32] but this impairment has not been replicated [34].	Careful assessment of side-effects in other areas of cognitive or physiological function. Detrimental effects are difficult to predict *a priori*, but should not be overlooked.
Joint effects of probiotics and prebiotics	What are the independent and interactive effects of prebiotics and probiotics?		Four-armed investigation comprising the following groups: probiotics + placebo, prebiotics + placebo, probiotics + prebiotics, placebo + placebo. This would also reveal whether prebiotics and probiotics differentially alter microbiome composition.
Strain specificity	Why do some strains of probiotic or prebiotic produce effects but not others?	Some psychobiotic strains produce effects while others produce partial or no effects 27, 38, 44. Furthermore, strains from the same family may differentially affect anxiety. For example, *Bifidobacterium longum* (B.) 1714 reduced depressive behaviour in the tail suspension test, while *Bifidobacterium breve* 1205 reduced anxiety in the elevated plus maze [153]. In another case, *Bifidobacterium longum* (B.) 1714 produced cognitive benefits that were not evident from *Bifidobacterium breve* 1205 [154]. In studies that show effects of mixtures of probiotics 35, 42, 152, it is unclear whether the outcome emerges from additive or synergistic interactions between bacterial families, or whether only some of the probiotics in the mixture are truly exerting effects.	Rigorous comparison of the effects of different strains, and efforts to replicate findings of strain-level differences from earlier studies. The development of a theoretical account of how and why certain different species and strains exert differing effects would enable specific predictions of which strains exert which effects under which conditions. Such a framework is currently lacking.
Role of moderators	What factors moderate psychobiotic effects?	Effects of *Lactobacillus helveticus* R0052 on inflammation and anxiety depend on diet and genotype [155]. Other potential moderators include age and sex.	Systematic efforts to identify and assess individual differences and other moderators that could have an effect on psychobiotic outcomes.
Drug interactions	How do psychobiotics interact with other psychotropic substances?		Clinical trials of the adjuvant therapeutic properties of psychobiotics alongside mainstream anxiolytics and antidepressants.

## Glossary

Colonisation: bacterial colonisation is believed to begin during parturition. Infant gut bacteria carry a maternal signature, though the mode of delivery substantially influences the composition of the early microbiota. The human infant's microbiome comes to resemble the adult microbiome in complexity and richness in the first 1 to 3 years.
Commensal gut bacteria: communities of indigenous bacteria residing in the intestinal tracts that share symbiotic relationships with the host. Bacteria comprise the vast majority of the microbiome, including at least 1000 distinct species and 7000 strains. Anaerobic bacteria are the chief residents of this community, which is composed mainly of *Firmicutes* and *Bacteroidetes* (up to 75%) along with smaller communities of *Actinobacteria*, *Fusobacteria*, *Proteobacteria*, and *Verrucomicrobia*. Alongside bacteria reside smaller numbers of archaea, bacteria, fungi, protozoa, and viruses.
Cytokine: proteins that facilitate cell–cell signalling. They serve an essential role in the organism's immune activity, and balance inflammation in response to pathogenic or infectious entities. Pro-inflammatory cytokines enhance inflammation, while anti-inflammatory cytokines suppress inflammation. Healthy immune function requires balanced pro- and anti-inflammatory cytokines. Depression and anxiety have both been associated with excessive pro-inflammatory cytokines.
Enteric nervous system: a subdivision of the nervous system embedded throughout the entire gastrointestinal tract, regulating all gastrointestinal function (e.g., gut motility, mucus secretion, blood flow, and immunological and endocrine activity).
Glucocorticoid stress response: a hormonal end-product of activity in the hypothalamic-pituitary-adrenal (HPA) axis. Glucocorticoids such as cortisol and corticosterone are well-validated biomarkers of stress. In particular, circulating glucocorticoid concentrations increase in response to psychological and physical stress. Their secretion alters glucose metabolism and inhibits immunological activity. They also increase threat sensitivity and negative mood, and impair memory and other cognitive functions. In humans, cortisol is the glucocorticoid that performs these functions. In rats and mice, corticosterone exerts these effects. Overall, though cortisol and corticosterone have somewhat different molecular structures, they are functionally similar in their physiological and psychological effects. While glucocorticoids prepare the organism to deal with stressful or uncertain situations, dysregulated HPA-activity and glucocorticoid levels are associated with anxiety and depression.
Gut–brain axis: a network comprising the gastrointestinal tract, the enteric nervous system, and the brain. Bidirectional communications between these entities regulate several important functions, including immunity, digestion, metabolism, satiety, and stress reactions.
Messenger ribonucleic acid: mRNA carries genetic information for the coding of particular proteins from the deoxyribonucleic acid (DNA) within the cell's nuclear envelope into the cytoplasm, where protein synthesis occurs.
Inflammation: an adaptive response to noxious stimuli or experiences, most notably infection or injury. The response involves coordinated activity across a range of biological actors, including cytokines. These processes are targeted at protecting tissue from further damage and repairing or clearing affected tissue.
Rodent behavioural tests: in animal models, psychological states such as anxiety and depression must be inferred from behavioural profiles. Various tests allow for the systematic observation of certain behaviours that correspond closely to the psychological state of interest. These tests have also been shown to accurately capture anxiolytic and antidepressant effects. In maze-learning tasks, rodents are exposed to novel environments. Over time, they develop spatial representations for the maze through attempts to navigate it. Anxiety impairs this learning process. The Morris water maze, in which rodents swimming in pools of water must find a submerged platform which allows them to stop swimming, tests similar processes by exploiting the evolutionary fear of drowning. In the elevated plus maze, an evolutionary tendency to prefer closed to open spaces is exploited on a maze with walled-in and no-wall components maintained at a substantial elevation from the ground, with anxious behaviour measured as preference for, and time spent in, the walled sections. In the open field test, rodents are placed in a novel and open area that they subsequently explore, with anxious behaviour marked by tendencies such as time spent away from the centre of the space. The forced swim test measures depressive behaviour by assessing the vigour with which rodents swim in an enclosed area, with periods of immobility or poor performance reflecting depressive behaviour. In the tail suspension test, rodents are suspended by their tails, and periods of immobility or other signs of insufficient struggle to escape the suspension are taken as markers of depression.
Rodent stress inductions: stress can be induced in rodents through exposure to noxious stimuli. These stressors enable experimental analysis of subsequent behavioural and physiological reactions. Restraint stress physically confines the rodent such that it is impossible to move. Water avoidance stress entails surrounding the rodent with water, which triggers the fear of drowning. A developmental approximation of anxiety and depression is achieved through maternal separation, in which infants are separated from their mothers shortly after birth. This exerts negative emotional and physiological effects throughout the rodent's developmental trajectory.
Waking cortisol response: cortisol rises continuously on first waking by approximately 50%. The precise purpose of this response is still unknown. It serves as a biomarker of HPA-reactivity, and is exaggerated in depression and anxiety.
